# Content Validity of a Short Calcium Intake List to Estimate Daily Dietary Calcium Intake of Patients with Osteoporosis

**DOI:** 10.1007/s00223-016-0221-8

**Published:** 2017-01-12

**Authors:** L. A. Rasch, M. A. E. de van der Schueren, L. H. D. van Tuyl, I. E. M. Bultink, J. H. M. de Vries, W. F. Lems

**Affiliations:** 1 Department of Rheumatology, Amsterdam Rheumatology and Immunology Center, Location VU University Medical Center, De Boelelaan 1117, 1081 HV Amsterdam, The Netherlands; 2 Department of Nutrition and Dietetics, VU University Medical Center, De Boelelaan 1117, 1081 HV Amsterdam, The Netherlands; 3 Division of Human Nutrition, Wageningen University, Bomenweg 2, 6703 HD Wageningen, The Netherlands

**Keywords:** Calcium, Dietary history, Osteoporosis, Questionnaire, Supplementation, Validation

## Abstract

**Purpose:**

Calcium supplements are prescribed for prevention of osteoporotic fractures, but there is controversy whether excess of calcium intake is associated with cardiovascular events. While an accurate estimation of dietary calcium intake is a prerequisite to prescribe the adequate amount of supplementation, the most adequate tools for estimating intake are time-consuming. The aim of this study is to validate a short calcium intake list (SCaIL) that is feasible in daily clinical practice.

**Methods:**

Based on the food groups contributing most to daily dietary calcium intake and portion sizes determined in an earlier study, a three-item, 1-min SCaIL was designed. As a reference method, an extensive dietary history (DH) with specific focus on calcium-rich foods and extra attention for portion sizes was performed. Beforehand, a difference of ≥250 mg calcium between both methods was considered clinically relevant.

**Results:**

Sixty-six patients with either primary (*n* = 40) or secondary (*n* = 26) osteoporosis were included. On average, the SCaIL showed a small and clinically non-relevant difference in calcium intake with the DH: 24 ± 350 mg/day (1146 ± 440 vs. 1170 ± 485 mg, respectively; *p* = 0.568). Sensitivity and specificity of the SCaIL, compared to the DH, were 73 and 80%, respectively. However, in 50% of the individuals, a clinically relevant difference of ≥250 mg calcium was observed between both methods, while in 17% this was even ≥500 mg.

**Conclusions:**

The SCaIL is a quick and easy questionnaire to estimate dietary calcium intake at a group level, but is not sufficiently reliable for use in individual patients. Remarkably, the mean dietary calcium intake estimated by the DH of 1170 mg/day indicates that a large proportion of osteoporosis patients might not even need calcium supplementation, although more data are needed to confirm this finding.

## Introduction

Calcium supplements are frequently prescribed for the prevention of osteoporotic fractures. However, the recent literature suggests that too much calcium supplementation may be associated with cardiovascular events. A five-year randomized controlled trial [1] and two meta-analyses [2, 3] of Bolland et al. demonstrated that use of calcium supplements was associated with an increased risk of cardiovascular events. In these studies, 500 mg/day of calcium or more was administered to the participants in the intervention group and a placebo to the control group, with a background intake of dietary calcium of around 850 mg/day. Also Li et al. [4], Pentti et al. [5] and Anderson et al. [6] found an increased risk of myocardial infarctions, coronary heart disease and coronary artery calcification, respectively, among users of calcium supplements. In contrast to these studies, Lewis et al. [7] found no evidence that calcium supplements were associated with an increased risk of cardiovascular disease after administrating 1200 mg/day of calcium or identical placebo tablets, next to a mean intake of around 950 mg of dietary calcium. Also in their meta-analysis from 2015, they did not found evidence that calcium supplementation with or without vitamin D increases the risk of coronary heart disease in elderly women [8], as did Paik et al. [9].

Although the literature is still inconsistent and inconclusive about the association between calcium supplements and cardiovascular events [10], the supposed cardiovascular side effects are frequently debated by health professionals as well as by patients. Since optimal calcium intake is crucial in the prevention and treatment of osteoporosis, calcium supplements are prescribed to patients with an insufficient dietary intake of calcium. According to national [11] and international [12–14] guidelines, the Recommended Dietary Allowances (RDA) of calcium is 1000–1200 mg/day for patients with osteoporosis. An accurate estimation of dietary calcium intake is necessary to adequately prescribe calcium supplements up to the recommended intake.

Several food frequency questionnaires (FFQs) for estimating dietary calcium intake have been designed for patients with osteoporosis specifically and validated in the last decennium [15–17]. However, for accurate estimation by physicians, and as a basis for adequate prescriptions, these FFQs are too time-consuming. To our knowledge, no short questionnaire for a reliable estimation of dietary calcium intake of osteoporosis patients by physicians is available. Our group has recently shown that an existing calcium intake list with three items is not a valid method to estimate calcium intake of osteoporosis patients [18]. Since we are in search of an easy, accurate and feasible method to estimate calcium intake of osteoporosis patients, we developed a new short, quick and easy calcium intake list that can be used by physicians to estimate dietary calcium intake of patients with osteoporosis validly in less than two minutes, in order to prescribe adequate amounts of calcium supplements to the patient.

The aim of this study is to validate this short calcium intake list (SCaIL) with an extensive dietary history (DH) as a reference method, to accurately estimate dietary calcium intake in patients with osteoporosis in daily clinical practice.

## Methods

### Study Population

This descriptive cross-sectional study included consecutive patients attending the outpatient rheumatology department of the Amsterdam Rheumatology and Immunology Center, location VU University Medical Center in Amsterdam, the Netherlands, for the treatment of primary or secondary osteoporosis. Patients were recruited between March and December 2013. Inclusion criteria for this study were: age of 18 years or older at inclusion and treatment with anti-osteoporosis agents by a rheumatologist. In addition, patients with secondary osteoporosis had to be diagnosed with a rheumatic disorder by a rheumatologist. Pregnant women, cognitively impaired persons or patients who do not speak the Dutch language were excluded from this study.

### Measurements

#### Calcium Intake

**Fig. 1:**
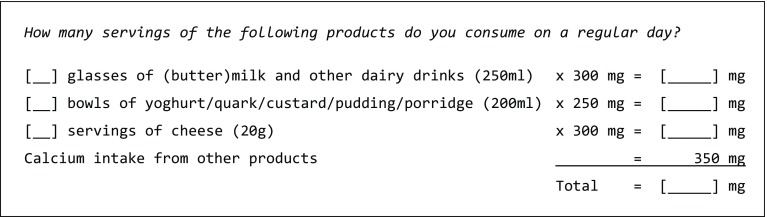
SCaIL: short calcium intake list to estimate daily dietary calcium intake of osteoporosis patients

The SCaIL is a short calcium intake list in which the patients are asked how many servings of dairy products they consume on a regular day (Fig. 1). The SCaIL was designed according to the main contributors to the daily dietary calcium intake using the outcomes of the Dutch National Food Consumption Survey 2007–2010 of the National Institute of Public Health and the Environment (RIVM) [19]. With the Dutch FFQ tool [20], the main contributors of calcium intake of a subgroup of Dutch adults aged 55–69 years were determined: (butter)milk and other dairy drinks (22.0%); yoghurt, quark, custard, pudding and porridge (12.8%); and cheese (29.0%). Together, these products contributed for 63.8% to the total dietary calcium intake of this subgroup of adults. These were the same foods as included in our previous, non-valid, calcium intake list [18]. Mean portion sizes of the foods included in the SCaIL were calculated using the mean portion sizes of the 66 osteoporosis patients who were included in the previous validation study [18]. To be able to calculate the calcium intake, the calcium content of each portion was calculated. Since the product groups in the SCaIL only represent 63.8% of total dietary calcium intake, a rest group was added to the SCaIL to reflect the amount of calcium intake from other foods. The rest group represents 36.2% (100 − 63.8) of the mean total dietary calcium intake of Dutch adults of 1040 mg [19]. This was rounded off to 350 mg to be able to easily sum up the individual calculations.

To be able to validate the SCaIL, an extensive DH with specific focus on calcium-rich foods and extra attention for assessment of portion sizes was performed as a reference method, after completing the SCaIL. A DH is a commonly used dietary assessment method to determine long-term, usual dietary intake of patients. It is a reliable reference method, since it takes into account day-to-day variation, and determines individual foods, frequency of food consumption as well as portion sizes [21]. Furthermore, to increase reliability, food consumption in a DH is asked per meal. Although this meal-based DH requires more time, patients can report their food consumption better per meal than over an entire day [21]. The researcher (LR) who performed all DHs in this study is also a dietician with experience in conducting DHs and probing, which is required to conduct a reliable DH. Since calcium was the main parameter, specific attention on calcium-rich foods was paid by performing a cross-check for these foods. Moreover, to improve estimation of portion sizes, samples of cups (100, 150, 220, 275, and 300 ml), glasses (100, 150, 200, 220, and 300 ml), bowls (100, 200, 250, and 450 ml) and slices of cheese (7, 10, 14, and 25 g) were used. Conducting and processing a DH took about 90 min per patient.

Beforehand, a difference of 250 mg calcium or more between the SCaIL and the DH was determined as clinically relevant. This amount was chosen because a difference of 250 mg/day or more may influence calcium prescription that usually is provided in units of 500 mg calcium.

#### Demographic and Clinical Characteristics

Demographic characteristics (age, gender, ethnicity, weight, height), lifestyle characteristics (smoking status, alcohol use), and disorder-related factors (previous clinical fractures) were assessed during the appointment with the researcher. From the medical charts, bone mineral density of the lumbar spine and total hip of the most recently performed dual-energy X-ray absorptiometry were obtained, as well as information about the use of medication and supplements. In addition, compliance of prescribed calcium and vitamin D supplements and use of extra ‘over the counter’ supplements on own initiative were checked by asking for details about supplements taken.

#### Statistical Analysis

Nutrient information of the DH was obtained using the Dutch nutrient database of NEVO online (version 2013/4.0, RIVM, Bilthoven, 2013) [22]. To calculate the amount of calcium, the nutrient calculation program Compl-eat (2010–2014 Human Nutrition, WUR, Wageningen, the Netherlands) was used. Data were analyzed using SPSS for Windows (IBM SPSS Statistics version 20, SPSS Inc., Chicago, IL, USA). Descriptive statistics were used to calculate the means, frequencies and percentages. When normally distributed, continuous data were compared using an independent sample *t* test, and the Mann–Whitney *U* test is used when data were not normally distributed. For categorical variables, a Chi-square test was used to compare means. To compare the mean difference in calcium intake between the SCaIL and the DH on group and individual level, paired *t* tests and the Bland–Altman plot were used. A one-sample *t* test was used to compare the mean calcium intake with the RDA of calcium and with the intake of Dutch adults in the Dutch National Food Consumption Survey 2007–2010. If not normally distributed, nonparametric tests were used instead of the *t* tests. Normally distributed continuous data are reported as mean ± standard deviations. Data which were not normally distributed are presented as median [interquartile range]. Categorical data are presented as frequency (%). Sensitivity and specificity were calculated, of the SCaIL compared to the DH for identifying patients with a daily intake of 1000 mg calcium or more and of 1200 mg calcium or more. Kappa (*к*) was used to calculate the agreement between the SCaIL and the DH to classify patients in the same category of calcium intake: low (<1000 mg/day) or sufficient (≥1000 mg/day). As arbitrary benchmarks for the strength of agreement, the following *к* values are used: *к* < 0 reflects ‘poor’ agreement, *к* = 0–0.20 ‘slight,’ *к* = 0.21–0.40 ‘fair,’ *к* = 0.41–0.60 ‘moderate,’ *к* = 0.61–0.80 ‘substantial’ and *к* = 0.81–1 ‘almost perfect’ agreement [23]. *p* values <0.05 were considered to be significant. The limits of agreement used for the Bland–Altman analysis were defined as mean difference ±1.96 standard deviations of the difference.

## Results

 In this study, 66 patients were included, of whom 40 patients had primary osteoporosis and 26 patients secondary osteoporosis associated with a rheumatic disorder. The demographic and clinical characteristics of the patients are depicted in Table 1.

**Table 1:** Demographic and clinical characteristics

	Total (*n* = 66)
Age (years)	65.8 ± 12.1
Female gender	57 (86.4)
Caucasian	59 (89.4)
Body mass index (kg/m^2^)^a,c^	24.4 [21.8–28.0]
*Disease-related factors*	
Lumbar spine (T-score)^a^	−1.76 ± 1.27
Total hip (T-score)^b^	−1.41 ± 0.78
Clinical fractures >25 years of age	43 (65.2)

Data are reported as mean ± standard deviation, median [interquartile range] or frequency (%)

^a^ 1 primary osteoporosis patient missing; ^b^ 2 primary osteoporosis patients missing; ^c^ 3 secondary osteoporosis patients missing

The mean ± SD dietary calcium intake was 1146 ± 440 mg/day by the SCaIL, and 1170 ± 485 mg/day by the DH. Consequently, the mean difference ± SD between both methods was 24 ± 350 mg/day, which was non-significant (*p* = 0.568) and beforehand determined as not clinically relevant (Table 2).

**Table 2:** Dietary calcium intake calculated using the SCaIL and using the dietary history method

	Calcium intake via SCaIL (mg/day)	Calcium intake via DH (mg/day)	Difference (mg/day)	*p* value
Total (*n* = 66)	1146 ± 440	1170 ± 485	24 ± 350	0.568
Primary osteoporosis (*n* = 40)	1173 ± 484	1220 ± 510	47 ± 346	0.391
Secondary osteoporosis (*n* = 26)	1104 ± 367	1094 ± 444	10 ± 359	0.884

Data are reported as mean ± standard deviation

*DH* dietary history, *SCaIL* short calcium intake list

**Fig. 2:**
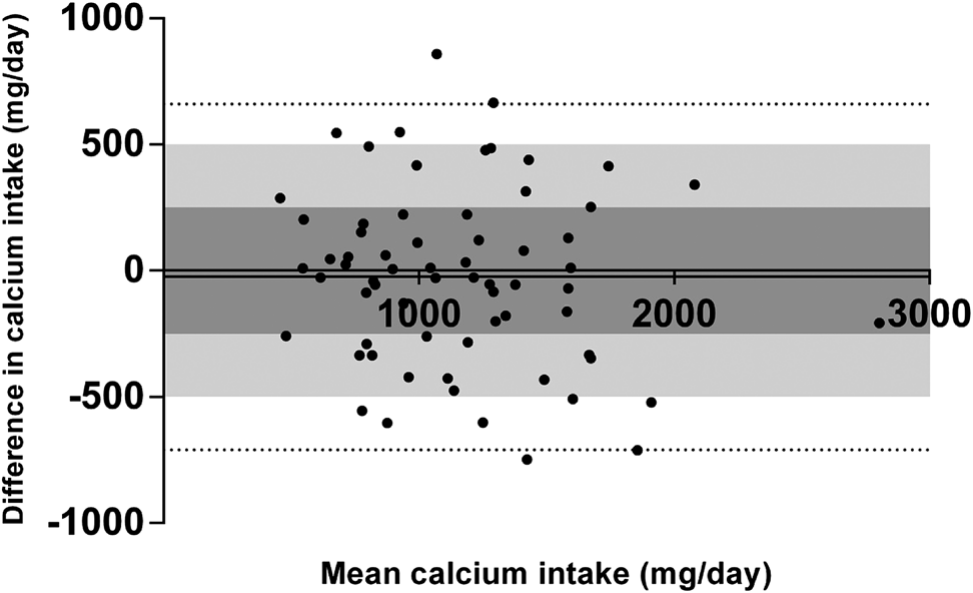
Bland–Altman plot of the mean and difference in calcium intake calculated using the dietary history method and the short calcium intake list. The *solid line* represents the mean difference in calcium intake (24.7 mg/day). The *dotted lines* are the limits of agreement defined as ±1.96 standard deviations of the difference. In *dark gray*, the area of clinically non-relevant difference in calcium intake (−250 to 250 mg/day) is displayed. In *light gray*, the area of 500 mg/day difference in calcium intake is displayed

Although the mean difference between the SCaIL and the DH was far smaller than 250 mg, a clinically relevant difference of 250 mg calcium or more was observed in 33 out of 66 patients (50%). Out of these 33 patients, the SCaIL underestimated calcium intake with ≥250 mg calcium in 19 patients (28.8%) and overestimated calcium intake with ≥250 mg calcium in 14 patients (21.2%), compared to the DH. A difference of ≥500 mg calcium was observed in 11 out of 66 patients (17%): the SCaIL underestimated calcium intake with ≥500 mg calcium in 7 patients (10.6%) and overestimated calcium intake with ≥500 mg calcium in 4 patients (6.1%), compared to the DH. These results are displayed as the Bland–Altman plot in Fig. 2. Sensitivity and specificity of the SCaIL compared to the DH were 73 and 80%, respectively, for identifying patients with a daily intake of 1000 mg calcium or more. For identifying patients with a daily intake of 1200 mg calcium or more, sensitivity and specificity of the SCaIL compared to the DH were 76 and 82%, respectively. The agreement between the SCaIL and the DH to classify patients in the same category, low calcium intake (<1000 mg/day) or sufficient calcium intake (≥1000 mg/day) was fair (*к* = 0.37).

The majority of patients (*n* = 52; 78.8%), had been prescribed 500 mg/day of calcium supplements by their treating physician. Of the remaining 14 patients, one patient (1.5%) had a prescription of 400 mg/day, eight patients (12.1%) had a prescription of 1000 mg/day and five patients (7.6%) did not have any prescription at all. Compliance to supplement prescription was on average 472 ± 218 mg/day. In addition, 15 patients used calcium containing ‘over the counter’ supplements on own initiative (mean in total population: 47 ± 143 mg/day). The mean total daily calcium intake (nutrition plus (all) supplements) amounted to 1689 ± 516 mg/day (Table 3). This is significantly higher than the RDA of 1000–1200 mg/day of calcium (*p* < 0.001). Four out of the 66 patients (6%) had a total daily calcium intake under the RDA of 1000 mg/day. Furthermore, also the mean dietary calcium intake of 1170 ± 485 mg calcium as assessed with the DH was significantly higher than the mean dietary calcium intake of healthy Dutch adults of 1040 mg (*p* = 0.033). Forty out of the 66 patients (61%) had a dietary calcium intake of 1000 mg or more and in addition a prescription for calcium supplements. In total, 18 out of the 66 patients (27.3%) had a total daily calcium intake above the tolerable upper intake level of 2000 mg/day.

**Table 3:** Total calcium intake divided into calcium intake through diet, through taken prescriptions and through over the counter supplements on own initiative

	Total (*n* = 66)
*Calcium intake*	
Diet (mg/day)	1170 ± 485
Prescribed supplements (mg/day)	472 ± 218
Over the counter supplements (mg/day)	47 ± 143
Total calcium intake (mg/day)	1689 ± 516

Data are reported as mean ± standard deviation

## Discussion

The SCaIL is a three-item, quick and easy questionnaire to estimate dietary calcium intake of osteoporosis patients at a group level. Mean calcium intake calculated with the SCaIL only differed 24 ± 350 mg/day of calcium with the DH, which was used as a reference method. Although these findings indicate that the SCaIL is a helpful tool to estimate mean dietary calcium intake in groups of osteoporosis patients, at an individual level a clinically relevant difference of 250 mg calcium or more was observed between the SCaIL and the DH in around half of the patients. This makes the SCaIL less useful for individual patients, because clinicians should be aware of not identifying a deficient calcium intake less than 1000 mg/day in approximately 20% of the patients and of prescribing supplements to patients with a sufficient intake in approximately 30% of patients, according to the sensitivity (80%) and specificity (73%) of the SCaIL compared to the DH.

Inaccuracy of the 1-min SCaIL for calculating dietary calcium intake in individual patients might most likely be attributed to the limited number of items to be recorded: Apparently, only three questions is not enough to be able to accurately estimate dietary calcium intake of osteoporosis patients. Since the three items in the SCaIL only represent 63.8% of total dietary calcium intake according to the Dutch National Food Consumption Survey, a rest group was added to reflect the amount of calcium intake from other products. However, the question remains whether the use of a standardized rest group is accurate (enough) to estimate the remaining dietary calcium intake, or that more detailed information on individual nutritional habits is necessary. This last option suggests that a questionnaire with more items would be more accurate. Indeed, existing validated FFQs for calcium contain more food items. However, as a consequence, it takes more time to fulfill those questionnaires, while that amount of time is not available in a doctor’s consulting room during busy clinical practice.

There are some limitations of this study. First, we used a commonly used dietary assessment method to calculate dietary calcium intake, however, not the ‘gold standard.’ For example, a food diary with weighted portion sizes over several days is considered to be a more accurate method to assess nutritional intake [24]. Since this method is very laborious and time-consuming for clinicians as well as burdensome for the patients, and therefore not frequently carried out on a large scale, we used a DH to calculate dietary calcium intake instead of the weighted food diaries, as a reference method to validate the SCaIL [21]. In this case, a FFQ would not be a preferred reference method since the SCaIL is a type of FFQ and assesses calcium intake more or less in the same way and therefore shows the same kind of errors [25]. Second, information bias could have occurred during the DH, since patients tend to give socially desirable answers, leading to a possible overestimation of actual calcium intake. However, this phenomenon is also likely to occur in other dietary assessment methods. Third, this study included ‘only’ 66 patients. However, our number of inclusions is similar to the numbers included in other recent studies validating calcium intake questionnaires [15–17], and we do not expect our results to be different if more patients were included. Fourth, we did not take into account the possibility that some of the patients do not use any dairy products. In our study population, this was limited to one patient. Food sources used by non-dairy consumers to replace the calcium intake of dairy products are not included in the SCaIL, since we especially looked at the main contributors of calcium intake of the general Dutch adult population to be able to focus on the general osteoporosis patient. Fifth, the total intake of calcium (nutrition plus (all) supplements), does not necessarily reflect intestinal calcium uptake, neither the amount of calcium incorporated in the skeleton. There are many factors affecting calcium metabolism, including intestinal calcium absorption and vitamin D levels. However, the estimation of intestinal or skeletal uptake of calcium is even more sophisticated, and not feasible to investigate in this study. Nevertheless, improvement in the estimation of dietary calcium intake is important to be able to prescribe the adequate amount of supplementation for more than one reason. First, as mentioned before, too much calcium supplementation possibly increases the risk of cardiovascular events. Second, when using calcium supplements, constipation, flatulence, diarrhea and nausea are regularly encountered side effects as well.

Remarkably, mean dietary calcium intake in our population is at the level of proposed current national and international recommendations. The national guidelines of the Dutch Institute for Health Care Improvement CBO [11] recommend a RDA of 1000–1200 mg/day of calcium, as do the international guidelines of the Institute of Medicine [20], the International Osteoporosis Foundation [21] and the National Osteoporosis Foundation [22]. In accordance with these guidelines, our population had a mean daily dietary calcium intake of 1170 ± 485 mg. Compared to the mean dietary calcium intake of Dutch adults (1040 mg/day [19]), the intake of our population is significantly higher (*p* = 0.033). Forty out of the 66 patients had a dietary calcium intake of 1000 mg or more and in addition a prescription for calcium supplements. Possibly, our patients are more aware of an adequate calcium intake in order to optimally treat their osteoporosis. Nevertheless, a considerably amount of patients (27.3%) had a total calcium intake above the tolerable upper intake level of 2000 mg/day. Since too much calcium supplementation may be associated with an increased risk of cardiovascular events, this is clinically unwanted and indicates that a large proportion of our osteoporosis patients might not even need calcium supplementation, although more data are needed to confirm this finding.

## Conclusions

 Although the SCaIL gives similar mean estimates of daily dietary calcium intake at a group level, it is not a valid method to estimate daily dietary calcium intake of individuals with osteoporosis. While aiming for enhanced feasibility through simplicity, this tool is unreliable for its intended purpose: the use in daily clinical practice in an outpatient clinic. Therefore, we do not advise to use the SCaIL in the consulting room of an outpatient clinic. More extensive FFQs take more time than available to fulfill, but as a result they provide a more accurate estimation of dietary calcium intake. The challenge remains to develop a new reliable calcium intake list with a balance between a limited number of items and a high accuracy of estimating dietary calcium intake. In the meantime, clinicians should be aware that a large proportion of the osteoporosis patients might not even need calcium supplementation because of a sufficient dietary intake, and those patients might be at increased risk of cardiovascular events while taking calcium supplements.
